# Cyclodextrin-Complexed *Ocimum basilicum* Leaves Essential Oil Increases Fos Protein Expression in the Central Nervous System and Produce an Antihyperalgesic Effect in Animal Models for Fibromyalgia

**DOI:** 10.3390/ijms16010547

**Published:** 2014-12-29

**Authors:** Simone S. Nascimento, Adriano A. S. Araújo, Renan G. Brito, Mairim R. Serafini, Paula P. Menezes, Josimari M. DeSantana, Waldecy Lucca Júnior, Pericles B. Alves, Arie F. Blank, Rita C. M. Oliveira, Aldeidia P. Oliveira, Ricardo L. C. Albuquerque-Júnior, Jackson R. G. S. Almeida, Lucindo J. Quintans-Júnior

**Affiliations:** 1 Laboratory of Pre-Clinical Pharmacology (LAPEC), Department of Physiology, Federal University of Sergipe, Av. Tancredo Neves, S/N, Rosa Elza, CEP: 49.000-100, São Cristóvão, Sergipe 49.100-000, Brazil; E-Mails: simonenascimento.saude@gmail.com (S.S.N.); guedes_renan@hotmail.com (R.G.B.); 2 Department of Pharmacy, Federal University of Sergipe, São Cristóvão, Sergipe 49.100-000, Brazil; E-Mails: adriasa2001@yahoo.com.br (A.A.S.A.); maiserafini@hotmail.com (M.R.S.); paulamenezes_16@yahoo.com.br (P.P.M.); 3 Department of Physical Therapy, Federal University of Sergipe, Aracaju, Sergipe 49.060-108, Brazil; E-Mail: josimelo@infonet.com.br; 4 Department of Morphology, Federal University of Sergipe, São Cristóvão, Sergipe 49.100-000, Brazil; E-Mail: wlucca1@gmail.com; 5 Department of Chemistry, Federal University of Sergipe, São Cristóvão, Sergipe 49.100-000, Brazil; E-Mail: periclesbalves@gmail.com; 6 Department of Agronomic Engineering, Federal University of Sergipe, São Cristóvão, Sergipe 49.100-000, Brazil; E-Mail: afblank@ufs.br; 7 Medicinal Plants Research Center, Federal University of Piauí, Teresina, Piauí 64.049-550, Brazil; E-Mails: menesesoliveira@gmail.com (R.C.M.O.); aldeidia@gmail.com (A.P.O.); 8 Institute of Technology and Research, University Tiradentes, Aracaju, Sergipe 49.032-490, Brazil; E-Mail: ricardo_luiz@itp.org.br; 9 Department of Pharmacy, Federal University of San Francisco Valley, Petrolina, Pernambuco 56.304-917, Brazil; E-Mail: guedesjackson@yahoo.com.br

**Keywords:** *Ocimum basilicum*, essential oil, β-cyclodextrin, pain, fos protein, fibromyalgia

## Abstract

*O. basilicum* leaves produce essential oils (LEO) rich in monoterpenes. The short half-life and water insolubility are limitations for LEO medical uses. β-Cyclodextrin (β-CD) has been employed to improve the pharmacological properties of LEO. We assessed the antihyperalgesic profile of LEO, isolated or complexed in β-CD (LEO/β-CD), on an animal model for fibromyalgia. Behavioral tests: mice were treated every day with either LEO/β-CD (25, 50 or 100 mg/kg, p.o.), LEO (25 mg/kg, p.o.), tramadol (TRM 4 mg/kg, i.p.) or vehicle (saline), and 60 min after treatment behavioral parameters were assessed. Therefore, mice were evaluated for mechanical hyperalgesia (von Frey), motor coordination (Rota-rod) and muscle strength (Grip Strength Metter) in a mice fibromyalgia model. After 27 days, we evaluated the central nervous system (CNS) pathways involved in the effect induced by experimental drugs through immunofluorescence protocol to Fos protein. The differential scanning analysis (DSC), thermogravimetry/derivate thermogravimetry (TG/DTG) and infrared absorption spectroscopy (FTIR) curves indicated that the products prepared were able to incorporate the LEO efficiently. Oral treatment with LEO or LEO-βCD, at all doses tested, produced a significant reduction of mechanical hyperalgesia and we were able to significantly increase Fos protein expression. Together, our results provide evidence that LEO, isolated or complexed with β-CD, produces analgesic effects on chronic non-inflammatory pain as fibromyalgia.

## 1. Introduction

Fibromyalgia is a chronic musculoskeletal disorder of unknown etiology characterized by chronic widespread pain, presence of tender points on physical examination, as well as symptoms that include fatigue, morning stiffness, sleep disorders and depression [1]. The pharmacological therapy currently recommended for fibromyalgia includes antidepressants, calcium-channel modulators, muscle relaxants and analgesics [2,3]. However, many patients fail to respond satisfactorily or have consistent side effects associated with these drugs in long-term use. Therefore, the control of chronic pain, as FM, remains as a challenge for medicine [4,5].

An important approach to discover new medicines is survey of natural products, such as medicinal plants or their secondary metabolites that modulate painful conditions [6]. Essential oils are extracted from various aromatic plants generally located in temperate to warm countries, like Brazil, where they represent an important part of the traditional pharmacopoeia due to their important biological activities [7,8,9]. Additionally, monoterpenes, mainly compounds of essential oils, are chemical entities that have attracted scientific interest due to the diversity of compounds with pharmacological properties applicable in pain management [10,11,12]. Several *Ocimum* species (Lamiaceae) are used to treat central nervous system disorders in various parts of the world and their anticonvulsivant, analgesic and anti-inflammatory activities are frequently reported [13,14,15]. Sweet basil (*O. basilicum*) is an herb used to add a distinct aroma, flavor to food and as remedy in folk medicine to treat anxiety, epilepsy and pain [13]. In this context, *O. basilicum* leaf essential oil (LEO) (access “Maria Bonita”) rich in monoterpenes, such as linalool [14,16] appears as an interesting alternative for the treatment of pain conditions.

Despite the pharmacological properties attributed to LEO, water insolubility is one limitation to the use of LEO for pharmacological applications. Consequently, several approaches have been employed to improve chemical and pharmacological properties of lipophilic compounds [17,18,19]. The host-guest complexes of pharmaceutical compounds with cyclodextrins (CDs) have been extensively studied and used to improve their solubility, dissolution rate and bioavailability of poor water-soluble drugs [20]. Recently, our group has shown that the formation of CDs-complex with essential oils or monoterpenes improves water solubility and increases bioavailability, technical features that limit the therapeutic use of essential oil and terpenoids [15,19,21]. Additionally, we demonstrated that complexation with linalool, the main compound of essential oil of *O. basilicum*, in β-cyclodextrin (β-CD) improved analgesic profile when compared with linalool alone, this effect seem to involve descending inhibitory pain pathways in fibromyalgia animal model [22].

Previous studies have demonstrated that several *Ocimum* species are used to treat central nervous system (CNS) disorders in various regions of the world, mainly in developing countries, and their analgesic profile is frequently reported [8,13]. However, the poor water solubility and short half-life of essential oils and related compounds, such as terpenes, have that otherwise limited their therapeutic use. Drug-delivery systems, such as cyclodextrins, have been used to increase aqueous solubility and bioavailability/stability of terpenes or essential oils [18,21,23,24]. Thus, the aim of this study was to evaluate the antihyperalgesic effects of LEO and LEO/β-CD in experimental non-inflammatory chronic muscle pain in mice (related to be an animal model for Fibromyalgia) [25,26,27], and investigate whether LEO/β-CD complex improves pharmacological activity of LEO isolated. We also evaluated a possible involvement of the central nervous system areas.

We also studied whether the β-CD can be able to improve the pharmacological profile of LEO. Up to the present date, this is the first study evaluating preclinical anti-hyperalgesic effect of LEO and LEO/β-CD in experimental fibromyalgia in animal model, besides the goal to elucidate the central nervous system areas involved in this activity by immunohistochemistry for c-fos protein, a useful marker for the control of neuronal activity of the central pathways, particularly in the pain pathway

## 2. Results and Discussion

### 2.1. GC-MS and GC-FID Analysis

The results in Table 1 demonstrate that GC-MS and GC-FID analysis of LEO resulted in the identification of 13 compounds, consisting 100% of the total oil. Furthermore, 68.96% of linalool, 13.09% of geraniol and 6.12% of 1.8% cineol were the main components, comprising 88.17% of LEO (Table 1).

**Table 1:** Volatile composition of leaf essential oil of *Ocimum basilicum*.

Peak	RT (min)	Compound	GC-MS (%)	RRI exp. ^b^	IRR ^c^
1	7.517	α-pinene	0.15	932	932
2	8.825	sabinene	0.14	971	969
3	8.992	β-pinene	0.51	977	975
4	10.942	1.8-cineole	6.12	1032	1026
5	13.542	linalool	68.96	1102	1095
6	17.033	α-terpineol	0.72	1194	1186
7	19.083	geraniol	13.09	1250	1249
8	20.317	isobornyl acetate	0.38	1284	1283
9	23.592	acetategeranyl	2.83	1377	1379
10	24.017	β-elemene	0.35	1389	1389
11	25.058	(*E*)-caryophyllene	0.27	1420	1417
12	25.483	trans-α-bergamotene	2.25	1433	1432
13	27.100	amorpha-4,7(11)-diene	0.83	1482	1479
14	27.767	α-bulnesene	0.20	1502	1509
15	28.125	γ-cadinene	0.86	1513	1513
16	31.333	NI ^a^	0.27	1616	NI
17	32.133	epi-α-cadinol	2.07	1643	1638

^a^ NI: Not identified; ^b^ RRI exp.: Relative retention index calculated using a homologous series of *n*-alkanes (C9–C18) in an apolar capillary column DB-5MS; ^c^ According to Adams (2007) [28].

GC-MS and GC-FID analysis of the LEO resulted in the identification of 13 compounds, with linalool, geraniol and 1.8 cineol as the major compounds. The LEO composition was similar as previously described by Oliveira *et al.* [13], Hence, 68.96% of LEO is comprised (−)-linalool. Thermal analyses of the LEO/β-CD particles revealed the LEO was complexed in the β-cyclodextrin (β-CD). The curves corresponding to LEO/β-CD complexes did not show a sharp endothermic peak in the range of the volatilization of the pure compound (150 °C). The disappearance of this event is due to its encapsulation in the host β-CD. Thus, the DSC curves of the LEO/β-CD complexes indicate endothermic peaks: the first in the range of 25–121 °C (which corresponds to the release of water molecules as well as the release of LEO, probable adsorbed in the surface), the second in the range of 121–270 °C, where LEO strongly encapsulated is released, and at ~280 °C, where the decomposition of CDs molecules appears. In the case of β-CD, only the peaks corresponding to the release of water molecules (higher than in the case of complexes) and to decomposition appear. Similar results were observed by Hădărugă *et al.* [29], who studied the influence of the hydrophobicity of solvent mixture and the preheating temperature on the water extraction process for α- and β-CD, as well as for their complexes with various essential oils using classical Karl Fischer titration method and thermogravimetric analysis.

### 2.2. Thermal Analyses

**Figure 1:**
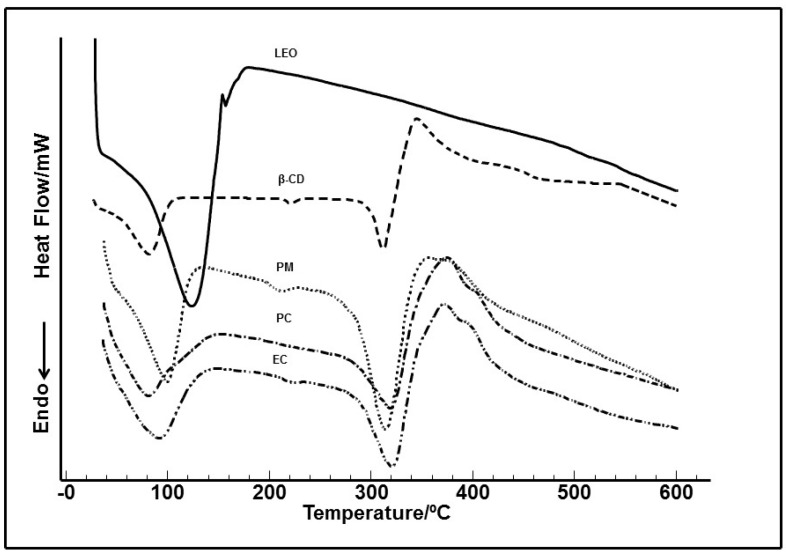
DSC curves of the essential oil of *Ocimum basilicum* (LEO), β-cyclodextrin (β-CD), physical mixture (PM), paste complex (PC) and co-evaporation (EC) obtained in a dynamic atmosphere of N_2_.

Thermal analyses of the LEO/β-CD particles revealed the formation of complexes. The DSC curves of LEO shows an endothermic peak at nearly 150 °C corresponding to its volatilization. As can be seen in Figure 1, the curves corresponding to LEO/β-CD complexes did not show a sharp endothermic peak in the range of the volatilization of the pure compound (150 °C) suggesting the complexation (Figure 1).

**Figure 2:**
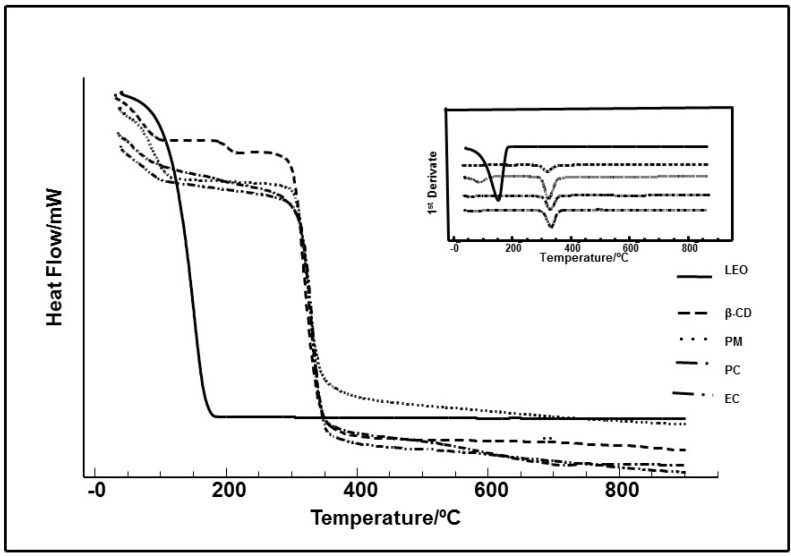
TG/DTG curves of the essential oil of *Ocimum basilicum* (LEO), β-cyclodextrin (β-CD), physical mixture (PM), paste complex (PC) and co-evaporation (EC) obtained in a dynamic atmosphere of N_2_.

As it can be seen in Figure 2, the results of physicochemical characterization DSC and TG/DTG curves indicated that the products prepared were able to incorporate the LEO efficiently by the co-evaporation technique, indicating the presence of the compound in the complex, what can increase the water solubility of the LEO (Figure 2).

### 2.3. Mechanical Hyperalgesia Analyses

**Figure 3:**
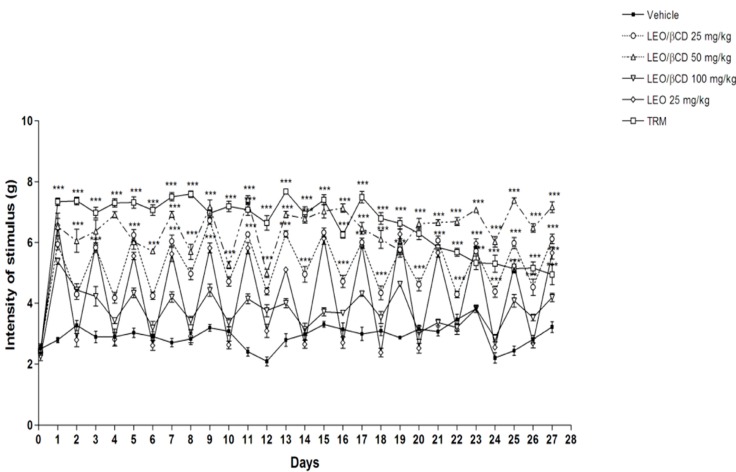
Effect of chronic administration of vehicle, *O. basilicum* essential oil (LEO; 25 mg/kg, p.o.), *O. basilicum* essential oil and β-Cyclodextrin (LEO-βCD; 25, 50 and 100 mg/kg, p.o.) or tramadol (TRM, 4 mg/kg) on mechanical hyperalgesia induced by acid saline. Each point represents the mean ± SEM of the paw withdrawal threshold (in grams) to tactile stimulation of the ipsilateral hind paw. *** *p* < 0.001 *vs.* control group (ANOVA followed by Bonferroni test).

The results in Figure 3 demonstrate that both LEO and LEO/β-CD complexes, at all tested doses, one hour beforehand, caused a marked inhibition of the nociceptive behavior (*p <* 0.05 or *p <* 0.001) in mechanical hyperalgesia. The anti-hyperalgesic effect induced by LEO/β-CD (25, 50 and 100 mg/kg) remained during 24 h, differently from the treatment with LEO isolated.

As expected, the pretreatment with tramadol (TRM, 4 mg/kg, i.p.), an opioid drug, caused a marked increase in the sensitivity threshold to mechanical stimuli (*p* < 0.001) and in all periods analyzed, according to the mechanical hyperalgesia assessment (Figure 3).

*In vivo* tests demonstrated that 24 h after the second injection of acidic saline into one gastrocnemius muscle, there was a significant decrease in mechanical withdrawal threshold of the paw bilaterally, which continued to gradually decrease until the 27th day. These data suggest that changes in the central nervous system maintain the bilateral, long-lasting hyperalgesia [24]. LEO-β-CD, at the lowest dose, produced persistent hyperalgesic effect when compared with LEO alone. This result shows that one of the benefits of using CDs complexation and the increased stability and bioavailability of oil or their main compounds, such as monoterpenes [15,18,19,30], is that one can associate with it. As expected, the treatment with tramadol (TRM 4 mg/kg, i.p.) caused a marked increase in the sensitivity threshold to mechanical stimulation (*p <* 0.001) at all analyzed periods according to assessment in the mechanical hyperalgesia. The variability in the 100 mg/kg treatment shown in Figure 3 suggests a probable phenomenon called hormesis. This paradoxical effect has been observed in a great variety of organisms, and with a large number of substances, poisons and drugs [31].

The antihyperalgesic effect demonstrated by LEO can be associated to the presence of monoterpenoids, such as (−)-linalool, which have a pronounced analgesic profile [11]. Several studies report the analgesic activity of (−)-linalool, which acts by modulating the muscarinic, opioid, dopaminergic, adrenergic and glutamatergic systems, and participating in ATP-sensitive K^+^ channels [14,32]. The synergistic effect of (−)-linalool exerts modulatory effect of pain through central and peripheral mechanisms [15,33], can provide benefits for the management of chronic pain syndromes such as fibromyalgia because it acts by different mechanisms and it can activate different pathways of the central nervous system.

In the fibromyalgia syndrome, myelinated afferent nerve fibers of the Aδ type obtain similar characteristics to those of C-type non-myelinated fibers, producing secondary pain [34]. Thus, a simple touch or pressure on the skin of the individual causes pain. Recent data showed that (−)-linalool, the major compound of LEO, blocks, in a concentration-dependent and reversible manner, the excitability and conduction of all types of myelinated fibers of the sciatic nerve with greater pharmacological potency for the fibers with slower conduction speed, blocking the generation of action potentials and inhibiting the voltage-gated Na^+^ current of dissociated dorsal root ganglion neurons [35]. Furthermore, it has been reported that the antinociceptive activity of LEO may have relationship with glutamate system, mainly due to the presence of linalool [14,32].

In the pathophysiology of fibromyalgia, there is participation of the descending inhibitory system, with the involvement of serotonergic, noradrenergic and opioid neurotransmission [36]. Furthermore, excitatory amino acids glutamate and aspartate play an essential role in nociception transmission through the spinal cord [37,38]. Therefore, LEO may be producing its effects due to its interaction with the glutamatergic and GABAergic systems [13].

In addition to increased neuronal mechanisms, glial cells also appear to play a role in the pathogenesis of FM, because they modulate pain transmission in the spinal cord. Activated by various painful stimuli, glial cells release proinflammatory cytokines, nitric oxide, prostaglandins and reactive oxygen species that stimulate and prolong spinal hyperexcitability [16]. In this way, monoterpenes, such as linalool and geraniol, can act synergistically and control the inflammatory response produced by glial cell activation, which has an important role in the pathogenesis of fibromyalgia. These cells, when activated by painful stimuli, are able to release proinflammatory cytokines, nitric oxide, prostaglandins and reactive oxygen species that stimulate and prolong spinal cord hyperexcitability [39].

### 2.4. Motor Coordination and Grip Strength Performance Analyses

**Figure 4:**
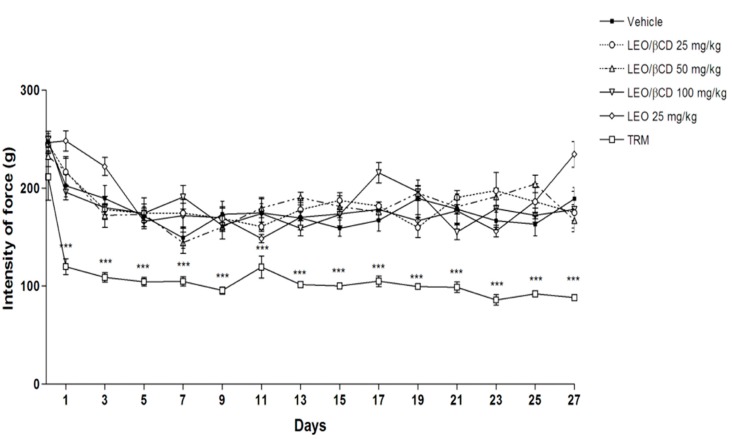
Effect of chronic administration of vehicle, *Ocimum basilicum* essential oil (LEO; 25 mg/kg, p.o.), *Ocimum basilicum* essential oil and β-Cyclodextrin (LEO-βCD; 25, 50 and 100 mg/kg, p.o.) or tramadol (TRM, 4 mg/kg) on grip strength test, fore-/hindlimb (4 paws). Bars represent the means (SEM) grip strength measurement (in grams of force) averaged across 3 trials. *** *p* < 0.001 *vs.* control group (ANOVA followed by Bonferroni test).

**Figure 5:**
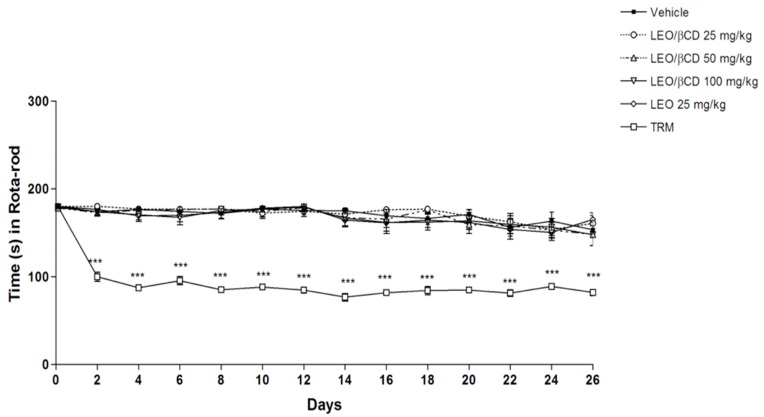
Effect of vehicle, *Ocimum basilicum* essential oil (LEO; 25 mg/kg, p.o.), *Ocimum basilicum* essential oil and β-Cyclodextrin (LEO-βCD; 25, 50 and 100 mg/kg, p.o.) or tramadol (TRM, 4 mg/kg) on the rota-rod test in mice. Values are the mean ± SEM (*n* = 8, per group). *** *p* < 0.001 *vs.* control group (ANOVA followed by Bonferroni test).

The present results demonstrated that all LEO or LEO/β-CD-treated mice, in the doses evaluated, did not have any performance alteration on the grip and rota-rod tests (Figure 4 and Figure 5).

Previous studies suggest that the central nervous system depression and the non-specific muscle relaxation effect can reduce the response of motor coordination and might invalidate the behavior test results [40]. Our results revealed that all mice treated with LEO or LEO-β-CD did not have any performance alteration in the Grip and Rota-rod apparatus, different from that observed in animals treated with TRM, reference drug.

### 2.5. Immunofluorescence Analyses

**Figure 6:**
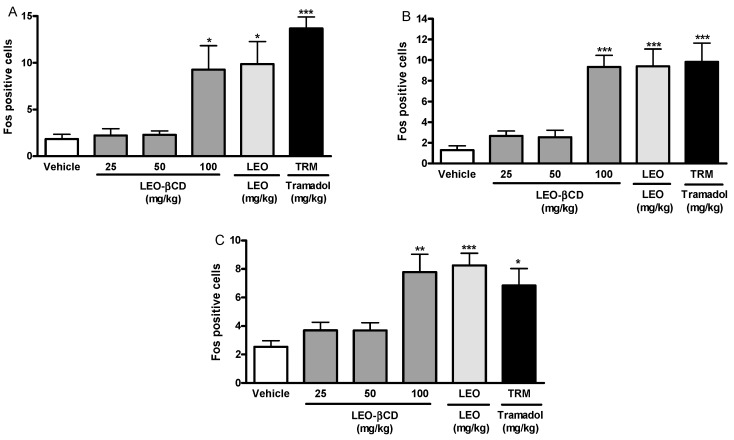
Neurons FOS positive in the periaqueductal grey (**A**); nucleus raphe magnus (**B**) and *locus coeruleus* (**C**). The animals were treated with vehicle (A; v.o.) LEO (B: 25 mg/kg; v.o.), LEO-β-CD (C: 25, D: 50 and E: 100 mg/kg; v.o.) or TRM (F: 4 mk/kg; i.p.). Values represent in mean ± SEM (*n* = 8, per group). * *p* < 0.05, ** *p* < 0.01 and *** *p* < 0.001 *vs.* control (one-way ANOVA followed by Bonferroni test).

The immunofluorescence for Fos protein showed that LEO and LEO/β-CD significantly activated (*p <* 0.05) neurons at PAG, NRM and LC, when compared with control (vehicle) animals in doses of LEO/β-CD 100 mg/kg, LEO 25 mg/kg and TRM (4 mg/kg) (Figure 6). However, the treatment with LEO and LEO/β-CD, at all doses, did not change the average number of neurons showing Fos protein in the gigantocelullar (GIG) and rostroventromedial medullary (RVM) when compared with control animals (data not shown).

**Figure 7:**
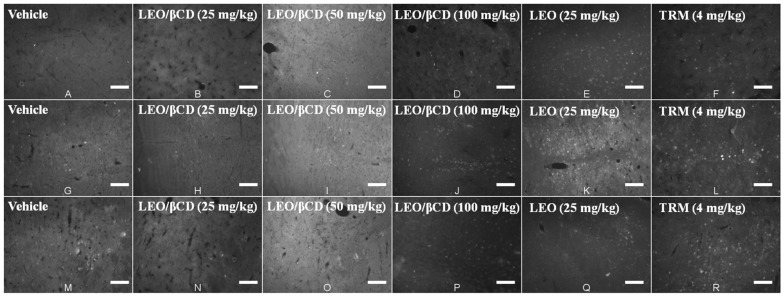
Immunofluorescence staining of nuclear Fos protein in the neurons of the periaqueductal grey (**A**–**F**); nucleus raphe magnus (**G**–**L**) and *locus coeruleus* (**M**–**R**), 90 min after the treatment with vehicle (v.o.); LEO (25 mg/kg; v.o.), LEO-β-CD (25, 50 and 100 mg/kg; v.o.) and TRM (4 mk/kg; i.p.).

The anti-hyperalgesic profile of LEO and LEO/β-CD can be mediated by the activation of the descending inhibitory pain pathways, evidenced by a significant increase in the number of Fos positive cells observed in the PAG, NRM and LC by the immunofluorescence technique using the protocol for Fos protein (Figure 7).

In order to demonstrate central nervous system areas activated by LEO (25 mg/kg) and LEO/β-CD (100 mg/kg), Fos protein labeled by imunofluorescence was performed in the present study, showing a significant activation of neurons at the periaqueductal gray (PAG), nucleus raphe magnus (NMR) and *locus coeruleus* (LC). However, the treatment with LEO/β-CD at doses of 25 and 50 mg/kg did not change the average number of neurons showing Fos protein in the Gigantocelullar (GIG) and Rostroventromedial Medullary (RVM).

The PAG region surrounds the cerebral aqueduct mesencephalic level and is a CNS area with great importance for the pain management. The PAG receives signals from the thalamus, hypothalamus, cortex and side connections of the spinothalamic tract, exciting the nuclei of the rostroventral medullary, like the raphe nuclei and the reticular formation. These areas are projected to the dorsal horn modulating the spinal nociceptive transmission of messages. In addition, the PAG, rostroventral medullary nuclei and dorsal horn of the spinal cord are targets of ventrolateral spinal axons, including the parabrachial nucleus and locus coeruleus [41].

Thus, the activation of the PAG, NRM and LC, observed in this study, exerts antinociceptive effect and inhibits responses of the spinal neurons [36]. Hence, the anti-hyperalgesic profile of LEO and LEO/β-CD, which can be mediated by the activation of the descending inhibitory pain pathways, suggests the involvement of CNS in the antihyperalgesic effect of LEO, probably by the modulation of opioid, noradrenergic and glutamatergic systems [33,35]. There is the participation of the descending inhibitory system in the pathophysiology of FM, with the involvement of serotonergic, noradrenergic and opioid neurotransmission [42].

## 3. Experimental Section

### 3.1. Plant Material and Essential Oil Extraction

Leaves were collected from cultivated *O. basilicum* L. plants at the Research Station “Campus Rural da UFS” (latitude 11°00'S and longitude 37°12'W) of the Federal University of Sergipe, Brazil. A voucher (Number # ASE15880) sample has been deposited in the Herbarium of the Department of Biology of Federal University of Sergipe. The leaves of *O. basilicum* were dried in an oven with air renewal and circulation (model MA-037/18) at 40 °C until complete dehydration. The essential oil was obtained through hydrodistillation in a Clevenger-type apparatus using 100 g of dry leaves, and dried with anhydrous sodium sulfate. Essential oil samples were analyzed by means of gas chromatography with flame ionization detector (GC-FID) coupled to mass spectrometry (GC-MS), using the Shimadzu^®^ GCMS-QP5050A apparatus (Shimadzu Europe, Duisburg, Germany). Each component was identified by comparing their mass spectra with spectra from the literature, with spectra evaluated by the database (NIST21 and NIST 107) equipment and by comparison of retention indices with those of the literature [43].

### 3.2. Preparation and Characterization of Inclusion Complexes

Both β-CD 98% and LEO, which were obtained from the extraction of oil from the leaves of *O. basilicum*, were used to prepare the samples. The complexation of LEO with β-CD was prepared through the co-evaporation technique (EC), in which β-CD (1135 mg) and LEO (154 mg) were mixed in molar ratio 1:1 (based on linalool molecular weight, the major component of the oil), in 20 mL of water under magnetic stirring at 400 rpm and kept constant for 36 h; after that, they were stored in a glass dessicator for ten days for drying the material. The products were characterized by means of differential scanning analysis (DSC), thermogravimetry/derivate thermogravimetry (TG/DTG) The TG/DTG curves were obtained, respectively, by means of thermobalanceTGA-51 and DSC-50 cell, using a heating rate of 10 °C/min. Assays TG/DTG were conducted in the temperature range of 25–900 °C under a dynamic atmosphere of N_2_ (50 mL/min), using capsule containing ~15 mg of sample. The DSC curves were obtained between 25 and 600 °C under a dynamic atmosphere of N_2_ (100 mL/min), using capsule containing Al 2 mg of sample. 

### 3.3. Animals

Experiments were performed on 48 male Swiss mice (25–35 g) obtained from the Animal Facilities of the Federal University of Sergipe. Mice were housed in controlled-temperature rooms (22–25 °C), under a 12:12 h light-dark cycle, with *ad libitum* access to water and food until use. All behavioral tests were performed between 8:00 a.m. and 2:00 p.m., and animals were used for 35 days. Experimental protocols were approved by the Animal Care and Use Committee at UFS/Brazil (CEPA 91/11). Every effort was made to minimize the number of animals used and any discomfort. All behavior experiments were performed under blind conditions to avoid influences of the observer in results.

### 3.4. Drugs and Reagents

Cyclodextrin (98% purity) was purchased from Sigma-Aldrich (St. Louis, MO, USA), TRAMADOL, in free form without additives, was purchased from Teuto/Pfizer (Anápolis-GO, Brazil, lote 00710/2012). Fluoromount G, glycine and bovine serum albumin (BSA) were purchased from Sigma (St. Louis, MO, USA). Ketamin and xylazin were purchased from Cristália (Itabira-SP, Brazil). Rabbit anti-Fos and donkey anti-rabbit Alexa Fluor 594 were obtained from Santa Cruz Biotechnology (Santa Cruz, CA, USA).

To prepare the pharmacological solution with LEO/β-CD before treatment, we used approximately 1:1 molar, as decribed by Quintans *et al.* [18] and Quintans-Júnior *et al.* [15]. Thus, when we administrated LEO/β-CD complex in mice, which was done in nominal dose. The LEO is not approved for use in human for the treatment of any disease, being in pre-clinical phase of test. All drugs were administered in volumes of 0.1 mL/10 g (mice), according to Batista *et al.* [43].

### 3.5. Behavioral Tests

#### 3.5.1. Acid Saline Induced-Chronic Muscle Pain

Before the first injection of acidic or normal saline into the gastrocnemious muscle, the animals had their paw withdrawal threshold evaluated in order to record the baseline value. Immediately after establishing the paw withdrawal threshold, animals of both groups were anesthetized with ketamine (60 mg/kg) and xylazin (80 mg/kg), and 20 µL of saline (pH 4.0) was injected in the left gastrocnemius muscle. This procedure was performed again 5 days after the first injection, and produced bilateral mechanical hyperalgesia lasting for 4 weeks after the second injection [23,25].

After confirming mechanical hyperalgesia, mice (*n* = 8, per group) were pretreated with LEO/β-CD, at the doses 25, 50 or 100 mg/kg; orally), LEO (25 mg/kg; orally), Tramadol (TRM 4 mg/kg; i.p.) or vehicle (Saline, orally), and 60 min after treatment they were screened for mechanical hyperalgesia through the digital von Frey, for motor coordination, through the Rota rod apparatus, and muscle strength through the Grip Strength Meter for 27 consecutive days, as previously described [23,25]. During this period, the animals received treatment every other day, starting on Day 1. After 27 days, we evaluated the central pathway involved in the effect through immunofluorescence protocol.

#### 3.5.2. Measurement of Mechanical Hyperalgesia

Mechanical hyperalgesia was tested in mice as reported by Cunha *et al.* [44], with adaptations by Guimarães *et al.* [45]. In a quiet room, mice were placed in acrylic cages (12 × 10 × 17 cm) with wire grid floors for 30 min before starting the test. This method consisted of evoking a hind paw flexion reflex with a hand-held force transducer (electronic analgesimeter; Model EFF 301, Insight^®^, Ribeirão Preto-SP, Brazil) adapted with a polypropylene tip. The investigator was trained to apply the tip perpendicularly to the central area of the hind paw with a gradual increase in pressure. The end point was characterized by the withdrawal of the paw followed by clear flinching movements. The intensity of the stimulus was obtained by averaging five measurements taken with minimal intervals of three minutes. In our experiments, all mice received the same pharmacological treatment as previously described in chronic muscle pain-induced acidic saline (pH 4.0).

#### 3.5.3. Measurement of Motor Coordination

A Rota-rod apparatus (AVS^®^, São Paulo-SP, Brazil) was used to assess whether the treatments with LEO or LEO/β-CD could influence the motor activity of the animals and consequently impair the assessment of the nociceptive behavior in experimental models [38]. The mice were pre-trained at a constant speed of 9 rpm once a day for 2 days before the test. Those mice that were able to remain on the rotating rod for 180 s were selected for the test. In our experiments, all mice received the same pharmacological treatment as previously described in chronic muscle pain-induced acidic saline. Performance time was assessed as the time until the three falls.

#### 3.5.4. Measurement of Forelimb Grip Strength

Immediately after the motor test, mice were assessed for grip strength performance. Grip strength performance was developed for use in rodent studies [22,46]; grip strength was measured as the tension force using the commercial grip strength meter (Insight^®^, Ribeirão Preto-SP, Brazil), which measures forelimb grip strength only. Mice were gently held by the base of their tails onto the top of the grid so that only their front paws were able to grip the grid platform/T-bar. The grip strength meter digitally showed the maximum force applied as the peak tensions (in grams) once the grasp was released. At this time, mice received the same pharmacological treatment as previously described in chronic muscle pain-induced acidic saline.

### 3.6. Immunofluorescence

To evaluate the action of the test drugs on the central nervous system, the animals were perfused and the brains were collected and cryoprotected for immunofluorescence processing to Fosprotein as described by Brito *et al.* [10] and Gama *et al.* [47]. Frozen serial transverse sections (20 μm) of all brains were collected on gelatinized glass slides. Tissue sections were stored at −80 °C until the use and were washed with phosphate buffer (0.01 M) saline isotonic (PBS) 5 times for 5 min and incubated with 0.1 M glycine in PBS for 10 min. Non-specific protein binding was blocked by incubation of the sections for 30 min in a solution containing 2% BSA. After that, the sections were incubated overnight with rabbit anti-Fos as primary antibodies (1:2000). Afterwards, the sections were incubated for two hours with donkey anti-rabbit Alexa Fluor 594 as secondary antibodies (1:2000). The cover slip was mounted with Fluoromount G. As an imunofluorescence control for non-specific labeling, sections were incubated without primary antibody. After each stage, slides were washed with PBS 5 times for 5 min.

A striking attribute of Fos is that it is a useful marker for the control of the neuronal activities of the central pathways of the sensorial system, particularly the nociceptive pathway. That protein can be detected in the neurons through immunohistochemical techniques from 20 to 90 min after the neuronal activation, disappearing from 4 to 16 h after the stimulus [10,47,48]. On account of that, we evaluated the action of the test drug on the central nervous system 90 min after the injection of the same pharmacological treatment as previously described in chronic muscle pain-induced acidic saline.

### 3.7. Acquisition and Analyses of Images

Pictures from Fos positive brain areas were acquired for each animal with an Olympus IX2-ICB (Olympus Group Companies, Tokyo, Japan). The areas of periaqueductal gray (PAG), nucleus raphe magnus (NRM), *locus coeruleus* (LC), gigantocelullar (GIG) and rostroventromedial medullary (RVM) were analyzed and classified according to Paxinus and Watsu Atlas (1997). Neurons were counted by the free software Image J (National Institute of Health, Bethesda, MD, USA) using a plug-in (written by the authors) that uses the same level of label intensity to select and count the Fos positive cells.

### 3.8. Statistical Analysis

Data obtained were expressed as the mean ± SEM and the differences were evaluated through one-way analysis of variance (ANOVA) followed by Bonferroni’s test. We considered as significant those with a value of *p* < 0.05. All statistical analyses were carried by means of the software GraphPad Prism 3.0 (GraphPad Prism Software Inc., San Diego, CA, USA).

## 4. Conclusions

We demonstrated that *O. basilicum* essential oil has an important anti-hyperalgesic profile, suggesting that this oil, isolated or complexed with β-CD, can be an interesting alternative for the development of new therapeutic options for the treatment of chronic painful conditions, as fibromyalgia. Ongoing studies allow us to understand precise mechanisms by which the LEO improves the bioavailability of the drug and increases the pharmacological effects.
